# Comparing the accuracy of the three popular clinical dehydration scales in children with diarrhea

**DOI:** 10.1186/1865-1380-4-58

**Published:** 2011-09-09

**Authors:** Kimberly Pringle, Sachita P Shah, Irenee Umulisa, Richard B Mark Munyaneza, Jean Marie Dushimiyimana, Katrina Stegmann, Juvenal Musavuli, Protegene Ngabitsinze, Sara Stulac, Adam C Levine

**Affiliations:** 1 Department of Emergency Medicine, Brown University Alpert Medical School, Providence, RI, USA; 2 Rwinkwavu District Hospital, Eastern Province, Rwanda; 3 Kirehe District Hospital, Eastern Province, Rwanda; 4 Butaro District Hospital, Northern Province, Rwanda; 5 Partners in Health/Inshuti Mu Buzima, Rwanda; 6 Rhode Island Hospital, 593 Eddy Street, Claverick 274, Providence, RI 02904, USA

## Abstract

**Background:**

Dehydration due to acute gastroenteritis is one of the leading causes of mortality in children worldwide. The World Health Organization (WHO) scale, the Gorelick scale, and the Clinical Dehydration Scale (CDS) were created to estimate percentage dehydration in children with gastroenteritis based on clinical signs. Of these, only the CDS has been prospectively validated against a valid gold standard, though never in low- and middle-income countries. The purpose of this study is to determine whether these clinical scales can accurately assess dehydration status in children when performed by nurses or general physicians in a low-income country.

**Methods:**

We prospectively enrolled a non-consecutive sample of children presenting to three Rwandan hospitals with diarrhea and/or vomiting. A health care provider documented clinical signs on arrival and weighed the patient using a standard scale. Once admitted, the patient received rehydration according to standard hospital protocol and was weighed again at hospital discharge. Receiver operating characteristic (ROC) curves were created for each of the three scales compared to the gold standard, percent weight change with rehydration. Sensitivity, specificity, and likelihood ratios were calculated based on the best cutoff points of the ROC curves.

**Results:**

We enrolled 73 children, and 49 children met eligibility criteria. Based on our gold standard, the children had a mean percent dehydration of 5% on arrival. The WHO scale, Gorelick scale, and CDS did not have an area under the ROC curve statistically different from the reference line. The WHO scale had sensitivities of 79% and 50% and specificities of 43% and 61% for severe and moderate dehydration, respectively; the 4- and 10-point Gorelick scale had sensitivities of 64% and 21% and specificities of 69% and 89%, respectively, for severe dehydration, while the same scales had sensitivities of 68% and 82% and specificities of 41% and 35% for moderate dehydration; the CDS had a sensitivity of 68% and specificity of 45% for moderate dehydration.

**Conclusion:**

In this sample of children, the WHO scale, Gorelick scale, and CDS did not provide an accurate assessment of dehydration status when used by general physicians and nurses in a developing world setting.

## Background

Diarrhea has the highest incidence of any childhood disease in all regions of the world and kills approximately 1.9 million children each year, accounting for 19% of all deaths in children under 5 [1,2]. The World Health Organization (WHO), the Centers for Disease Control and Prevention (CDC), the American Academy of Pediatrics (AAP), and the European Society of Pediatric Gastroenterology and Nutrition (ESPGAN) all support rehydration with oral rehydration solution (ORS) for children with mild to moderate dehydration, reserving intravenous (IV) fluids for children with severe dehydration [3-6]. Oral rehydration solution is a hypo-osmolar solution composed of salts, sugar, and citrate, while the recommended intravenous fluids include lactated Ringer's or normal saline [4,6-8].

In order to apply the most appropriate treatment for dehydration in children with gastroenteritis, healthcare providers must first accurately assess the severity of dehydration [5]. Underestimating fluid deficit, and not providing proper rehydration with either ORS or IV therapy can lead to acidosis, electrolyte disturbances, acute kidney injury, or even death. Alternatively, overestimating fluid deficit can lead to unnecessary interventions, longer hospital stays, and increased adverse events in children [9,10]. Accurate fluid assessment is of utmost importance in low- and middle-income countries, where many patients travel several hours to reach a healthcare facility, and resources such as IV fluids and hospital beds are scarce.

Several organizations and research institutions have developed scales to estimate dehydration status using clinical signs. The most popular are the WHO scale, the Gorelick scale (created at the Children's Hospital of Philadelphia), and the Clinical Dehydration Scale (CDS; created at the Hospital for Sick Children in Toronto) (please see Tables 1, 2, and 3 for scales). The scales predict percent dehydration for slightly different age groups; the CDS is for children between 1 month and 3 years, while the other two scales are for children between 1 month and 5 years. The WHO scale classifies children by grouping severity of symptoms, while the CDS has a scoring system. The Gorelick scale uses binary categorization of symptoms either as no dehydration or moderate/severe dehydration, and severity is determined by the number of physical signs present. Each scale also predicts a slightly different range for percent volume loss.

**Table 1:** WHO Scale for dehydration for children 1 month-5 years old

	A	B	C
Look at condition	Well, alert	Restless, irritable	Lethargic or unconscious
Eyes	Normal	Sunken	Sunken
Thirst	Drinks normally, not thirsty	Thirsty, drinks eagerly	Drinks poorly or not able to drink
Feel: Skin pinch	Goes back quickly	Goes back slowly	Goes back very slowly

Scoring: Fewer than two signs from column B and C: no signs of dehydration < 5%, ≥2 signs in column B: Moderate dehydration 5-10%, ≥2 signs in column C: > 10% severe dehydration

**Table 2:** The 10- and 4-point Gorelick Scale for dehydration: for children 1 month-5 years; 4-point scale physical exam signs highlighted in italic font

Characteristic	No or minimal dehydration	Moderate to severe dehydration
*General appearance*	*Alert*	*Restless, lethargic, unconscious*
*Capillary refill*	*Normal*	*Prolonged or minimal*
*Tears*	*Present*	*Absent*
*Mucous membranes*	*Moist*	*Dry, very dry*
Eyes	Normal	Sunken; deeply sunken
Breathing	Present	Deep; deep and rapid
Quality of pulses	Normal	Thready; weak or impalpable
Skin elasticity	Instant recoil	Recoil slowly; recoil > 2 s
Heart rate	Normal	Tachycardia
Urine output	Normal	Reduced; not passed in many hours

Scoring: 4 point scale (italics): ≥ 2 Clinical Signs (4 pt) ≥5% BWΔ; ≥

3 Clinical Signs (4 pt) ≥10% BWΔ; 10 point scale (all signs/symptoms):

≥ 3 Clinical Signs ≥5% BWΔ; ≥ 7 Clinical Signs ≥10% BWΔ

**Table 3:** CDS scale clinical features for prediction dehydration in children 1-36 months

Characteristic	0	1	2
General appearance	Normal	Thirsty, restless, or lethargic, but irritable when touched	Drowsy, limp, cold, sweaty, and/or comatose
Eyes	Normal	Slightly sunken	Very sunken
Mucous membranes	Moist	"Sticky"	Dry
Tears	Tears	Decreased tears	Absent tears

Scoring: 0: no dehydration < 3%, 1-4: some dehydration ≤3 × < 6%, 5-8: moderate dehydration ≥6%

Thus far, only the CDS has been prospectively validated against the accepted gold standard of percent weight change with rehydration at a single pediatric referral center in North America [11]. None of these scales have been validated in low- or middle-income countries where disease patterns may differ from high-income countries, patients often present later in the course of their disease, and healthcare providers often lack specialty training. The purpose of this study was to determine the accuracy of the WHO scale, Gorelick scale, and CDS in a resource-limited setting.

## Methods

### Study design

In this study we enrolled a non-consecutive cohort of children presenting with symptoms of diarrhea and/or vomiting to one of three district hospitals in Rwanda. The study was approved by both the Partners Healthcare (Massachusetts General Hospital) Institutional Review Board and the Rwanda National Ethics Committee. The child's parent or guardian provided either written on verbal consent in the local language, Kinyarwanda.

### Study setting and population

All three district hospitals, Kirehe, Rwinkwavu, and Butaro, serve rural and relatively impoverished populations. We enrolled children less than 15 years of age, the upper limit for admission to the pediatrics ward at each study hospital, but limited our analysis to children fitting within the predefined age ranges for each of the clinical dehydration scales. Each hospital has 25-40 inpatient pediatric beds and serves an estimated catchment area of 150,000 to 350,000 people. Enrollment occurred March-July 2009, and included all pediatric patients presenting with diarrhea and/or vomiting on weekdays from 7:00 a.m. - 5:00 p.m., and occasional nights and weekends based on availability of study staff.

### Data collection and methods of measurement

When an eligible patient presented to one of the three district hospitals, the nurse or physician caring for the patient contacted the local study coordinator. The coordinator then explained the study and obtained written or verbal consent in Kinyarwanda. The child was then weighed on a standard scale. The physician or nurse admitting the patient noted demographic information, nutritional status, and the signs and symptoms of dehydration.

Once admitted, the patient was treated according to standard hospital protocols, based largely on WHO protocols for management of dehydration in children. After they underwent rehydration therapy, the patient was weighed again on the same scale, and a discharge weight was recorded.

### Data analysis

First, basic descriptive statistics were calculated for our study population. We then took each of the clinical scales - WHO, Gorelick, and CDS - and classified each patient according to the scale. Receiver operating characteristic (ROC) curves were created for each of the three scales compared to the gold standard, percent weight change with rehydration. Percent weight change with rehydration was calculated by (rehydration weight - admission weight)/rehydration weight × 100%. Sensitivity, specificity, and likelihood ratios were calculated based on the best cutoff points for each of the ROC curves.

For the WHO and Gorelick scales, separate ROC curves were created for severe and moderate dehydration. For the CDS, ROC curves and sensitivities and specificities were created only for those children classified as having moderate dehydration (≥6% dehydration). All statistical analyses were performed using SPSS 16.0 (SPSS Inc., Chicago, IL).

## Results

Seventy-three children were enrolled in the study. Two children died prior to discharge, 12 children had evidence of severe malnutrition, and 7 children were missing discharge weights, leaving 52 children for analysis. Of these 52 children, 49 children were between 1 month and 5 years old and could be classified by the Gorelick and WHO scale, while 48 children were between 1 month and 3 years old and could be classified by the CDS.

The mean age was 10.5 months for children 1-36 months and 11.14 months for children 1 month-5 years. The median percent weight change for children between 1 and 36 months and children between 1 and 60 months was 4.8%. Twenty-nine percent of children presented with severe dehydration, classified as > 10% weight change between admission and discharge. The median length of stay was 4 days, with 88% of patients staying at least 3 days for both age groups.

**Figure 1:**
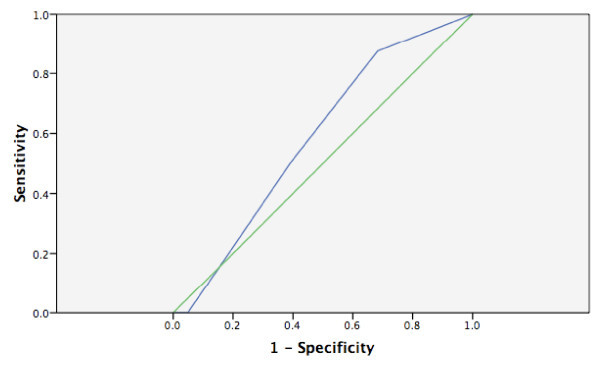
**WHO scale predicting moderate (5-10%) body weight change**.

**Figure 2:**
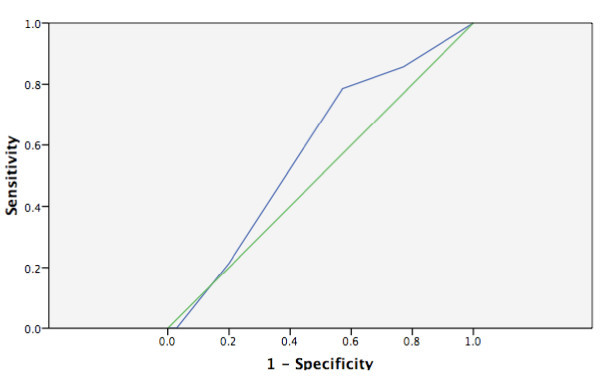
**WHO scale predicting severe (> 10%) body weight change**.

**Figure 3:**
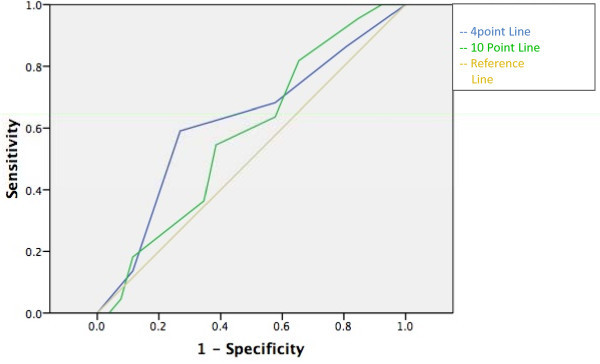
**Gorelick 4- and 10-point scale predicting moderate (≥5%) body weight change**.

**Figure 4:**
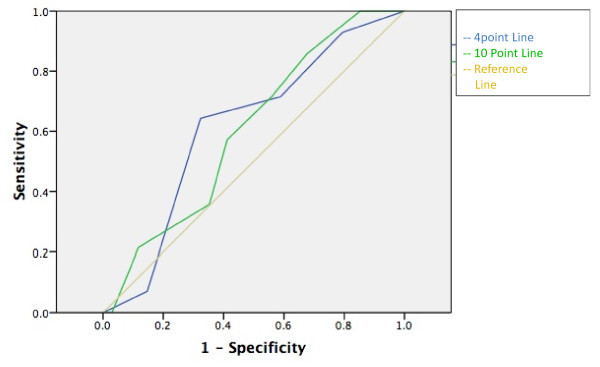
**Gorelick 4- and 10-point scale predicting severe (≥10%) body weight change**.

**Figure 5:**
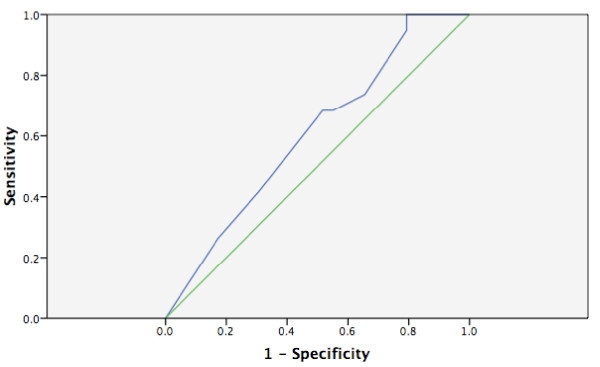
**CDS predicting moderate (≥6%) body weight change**.

None of the scales had an area under the ROC curve statistically different from the reference line (see Figures 1, 2, 3, 4 and 5). Stratifying by healthcare provider (doctor versus nurse) did not improve the accuracy of any of the ROC curves (data not shown). The WHO scale for moderate dehydration (5-10% percent weight change) had an area under the curve of 0.58 (95% CI = 0.39-0.78); sensitivity was 50% and specificity 61%; LR+ was 1.28 and LR- 0.82. For severe dehydration (> 10% percent body weight change) the WHO scale had an area under the curve of 0.58 (95% CI = 0.41-0.75); sensitivity was 79% and specificity 43%; LR+ was 1.38 and LR- 0.50. For moderate dehydration (between 5-10% percent body weight change) the 4- and 10-point Gorelick scales had an area under the curve of 0.62 (95% CI = 0.45-0.78) and 0.58 (95% CI = 0.42-0.74), respectively; sensitivities were 64% and 21%, and specificities were 69% and 82%, respectively; LR+ was 1.15 and 1.25 and LR- 0.78 and 0.53, respectively. For severe dehydration (≥10% body weight change) the 4- and 10-point Gorelick scale had areas under the curve of 0.62 (95% CI = 0.45-0.79) and 0.60 (95% CI = 0.44-0.77), respectively; sensitivities were 68% and 82%, and specificities were 41% and 35%, respectively; LR+ was 1.09 and 2.04, and LR- 0.52 and 0.88, respectively. For the prediction of moderate dehydration (percent weight change ≥6%), the CDS had an area under the curve of 0.64 (95% CI = 0.44-0.77). The sensitivity of the scale was 68% and the specificity 45%, with a LR+ of 1.24 and LR- of 0.70.

## Discussion

While most experts agree that children with diarrhea should be treated based on the severity of their dehydration, with children receiving intravenous fluids for severe dehydration and oral rehydration solution for mild to moderate dehydration, there is no clear consensus on how best to determine the severity of dehydration, especially in resource-limited settings. The gold standard for dehydration is percent volume loss with diarrhea, which is defined as the difference between pre-illness weight and acute-illness weight divided by pre-illness weight. Because pre-illness weight from a pediatrician's office is often not available, especially in low- and middle-income countries, it is ideally substituted by stable post-rehydration weight or the child's weight after undergoing therapy. In fact, Gorelick et al. validated post-rehydration weight as a surrogate for pre-illness weight by demonstrating near perfect correlation (*r *= 0.9988) between the two values in a small cohort of children with diarrhea [12]. In our study, we were not able to guarantee that participants had reached a stable post-rehydration weight prior to discharge. However, most children in the study by Gorelick et al. achieved a stable weight after 24 h in the hospital, and nearly all children achieved a stable weight by 72 h. Since all patients in our study spent at least 24 h in the hospital, and 88% spent more than 3 days, it is likely that they had the opportunity to achieve a stable rehydration weight, so we believe that percent weight change with rehydration can be used as a valid gold standard for dehydration in our study.

While percent weight change with rehydration makes an excellent gold standard for the severity of dehydration, it is not a useful tool in practice, since it is not available at the time of presentation when a decision about how best to manage a child with diarrhea must be made. For many years, experts have recommended the use of physical exam signs to predict the severity of dehydration in children with diarrhea. Steiner et al. found in a systematic review that the most useful individual signs for predicting 5% dehydration were abnormal capillary refill time, abnormal respiratory pattern, and abnormal skin turgor, which had positive likelihood ratios spanning from 2.0-4.1 [9]. However, none of these signs had very good negative likelihood ratios, meaning that they were not useful for excluding severe dehydration in children. Steiner et al. concluded that no individual clinical sign had adequate sensitivity and specificity for the prediction of dehydration. Other studies that have looked at laboratory values, such as BUN, anion gap, base deficit, bicarbonate concentration, and urine specific gravity, have generally not found them to be very good predictors of dehydration status, with only bicarbonate greater than 15 or 17 mEq/L useful for reducing the likelihood of dehydration [6,13-16].

Given the limitations of individual clinical signs, several prior authors have tried to combine physical findings into clinical scales to predict percent dehydration in children. Gorelick created a 4-point and 10-point scale for assessing dehydration in children 1-60 months old presenting to Children's Hospital of Philadelphia, resulting in sensitivities of 79% and 87% and specificities of 82% and 85%, respectively, for predicting ≥5% dehydration. The two scales had sensitivities of 82% and 90% and specificities of 83% and 90%, respectively, for ≥10% dehydration. The CDS was derived at the Toronto Hospital for Sick Children and then prospectively validated at that site by specialized pediatric staff. In the validation study, Parkin et al. demonstrated likelihood ratios for moderate dehydration of 2.2, 1.3, and 5.2 for CDS scores of 0, 1-4, and 5-8, respectively [11,17].

Neither the Gorelick scale nor the CDS performed as well in our population of children in Rwanda as they did in North America. In fact, both scales had areas under the ROC curves statistically indistinguishable from the reference line, meaning they were no better than chance at predicting moderate or severe dehydration. In addition, the WHO scale, considered the standard of care in most low- and middle-income countries, although it has never been prospectively validated for predicting severe dehydration, also performed poorly in our population of children. To our knowledge, this study is the first to prospectively assess a clinical dehydration scale in a low-income country, where children tend to present with more severe dehydration and be evaluated by personnel with less specialized training than their high-income country counterparts. Our study highlights the need for more research into better methods for detecting the severity of dehydration in children with diarrhea in resource-limited settings and supports a general rule that clinical scales derived in a high-income country setting require validation in resource-limited settings before being recommended for widespread use in these settings.

### Limitations

Study subjects were a convenience sample based on investigator availability; overall, we enrolled about half of eligible patients. Our sample size was small, but powered to detect a negative likelihood ratio less than 0.1 and a positive likelihood ratio greater than 2. A priori, we had decided that for a clinical scale to be useful, it had to reduce the likelihood of severe dehydration by at least a factor of 10 when negative (so as not to miss any children who truly need IV fluids) while at least doubling the likelihood of severe dehydration when positive (so as to not to result in the wasted resources and adverse events that come from over-treating children with IV fluids who do not actually have severe dehydration). Essential data were missing for about 10% of enrolled patients, who were therefore excluded from analysis. In addition, only children admitted to the hospital were enrolled in our study in order to be able to obtain both pre- and post-rehydration weights for the purpose of determining the gold standard of percent weight change with rehydration. Therefore, the children enrolled in our study were likely more ill than the average child with diarrhea and/or vomiting in a low-income country setting, limiting the generizability of our results. We attempted to include only children who would have weight change based on rehydration by excluding those children presenting with severe malnutrition who would receive dietary supplementation. It is unlikely that children not receiving dietary supplementation would have gained weight from improved nutrition because 87% of children in our study spent less than 1 week in the hospital, so it is unlikely that they would have been able to gain a significant amount of protein-energy weight in that time period while being fed a standard Rwandan diet.

## Conclusion

In this study, we found that the WHO scale, Gorelick scale, and CDS, when performed by general practice physicians and nurses in a resource-limited setting, were not accurate predictors of severe dehydration in children with diarrhea and/or vomiting. Due to the high prevalence and significant morbidity associated with diarrhea in children throughout the world, further research is necessary to develop and validate new clinical scales or other diagnostic tools with greater accuracy for assessing dehydration in children in resource-limited settings.

## List of abbreviations

CDS: clinical dehydration scale; ROC curve: receiver operating characteristic curve; LR: likelihood ratio; WHO: World Health Organization.

## Competing interests

The authors declare that they have no competing interests.

## Authors' contributions

KP analyzed data and wrote the manuscript. SPS developed the study concept and design, and participated in the acquisition of data. IU, RBMM, JMD, KS, JM, and PN participated in data acquisition. SS provided administrative, technical, and material support. AL conceived the study, participated in its coordination, and edited the manuscript.
